# Identification of Prognostic Values of Neutrophil Extracellular Traps‐Related Genes in Glioma Based on Bioinformatics

**DOI:** 10.1002/iid3.70422

**Published:** 2026-04-06

**Authors:** Xiaobing Guo, Xiaowen Li, Hengxi Li, Yan Cao, Pengfei Zhang, Ping Li

**Affiliations:** ^1^ Department of Anatomy and Histology & Embryology, Faculty of Basic Medical Science Kunming Medical University Kunming Yunnan China; ^2^ College of Sports and Health Yibin University Yibin Sichuan China

**Keywords:** ALDH1A3, CHCHD10, glioma, neutrophil extracellular traps (NETs), NFIL3

## Abstract

**Background:**

Glioma is a highly invasive and drug‐resistant malignant primary tumor. Increasing research is focusing on the function of neutrophil extracellular traps (NETs) in glioma progress. We aimed to explore the mechanism of NETs‐related genes (NETs‐RGs) in glioma to find potential biomarkers for glioma.

**Methods:**

The GSE16011 data set was downloaded from the GEO database, and the gene expression matrix and clinical data of glioma patients were downloaded from the TCGA database, the cbioportal website, and the CGGA database, as the training and validation sets. The NETs‐RGs were obtained from previous studies. Subsequently, differential expression analysis, WGCNA, GO enrichment, and GSEA analysis. The risk model was established for Cox, LASSO, survival, and independent prognostic analyses. The CIBERSORT algorithm was used for immune infiltration analysis, and pRRophetic was used for drug sensitivity analysis. Finally, the expression levels of genes were validated by data set, glioma patients' tissue samples, and glioma cells, and evaluating cell biological behavior.

**Results:**

A total of 57 differential expression genes between Glioma and Normal samples were obtained. Then, two modules with the highest positive correlation with NETs‐RGs by WGCNA, the NETs‐RGs were obtained from previous studies. Six candidate genes were obtained for subsequent analysis. Then, we conducted functional enrichment of candidate genes and constructed a glioma prognosis model. The prognosis model was indicated as a good predictor of a patient's glioma risk. These genes were related to immune cells significantly. And drug sensitivity analysis predicted 128 differences in chemotherapy drugs and found that MICALL2 had a significant correlation with multiple drugs. Finally, only NFIL3 had the same trend of significantly high expression levels. Moreover, knockdown NFIL3 can inhibit glioma cell malignant growth, and promote apoptosis.

**Conclusion:**

Three prognosis‐related genes have better prognosis values for glioma patients and may be the potential biomarkers for the treatment of glioma.

## Graphical abstract

Increasing research is focusing on the function of neutrophil extracellular traps (NETs) in glioma progress. However, the relationship between NETs and glioma development is not well understood. This study is based on the NETs‐related genes (NETs‐RGs), used WGCNA to analyze and identify the NETs‐RGs in glioma, and used a range of bioinformatics methods to evaluate the underlying molecular mechanisms of NETs‐RGs as the biomarker in glioma.

## 1 Introduction

Glioma is one of the common malignant primary tumors and also the main cause of death in brain cancer [1]. The grading of gliomas is based on their degree of malignancy and follows the World Health Organization (WHO) classification system, which comprises four grades. Grade 1 is a benign tumor with slow growth and a favorable prognosis. Grade 2 is a junctional tumor with a risk of recurrence and escalation. Grade 3 is moderately malignant, highly aggressive, and prone to recurrence. Grade 4 is highly malignant with rapid growth and a very poor prognosis. Molecular research plays a crucial role in tumor diagnosis. The fifth edition of the WHO Classification of Tumors of the Central Nervous System (CNS) (2021) incorporates numerous molecular alterations of clinical and pathological significance, further advancing the beneficial role of molecular diagnostics in CNS tumor classification [2]. The selection of study samples includes Grades 2 and 3 gliomas, which offer a rich source of research material due to their distinctive biological characteristics and clinical manifestations [3, 4]. The NCCN Guidelines for CNS have indicated that glioma is a heterogeneous tumor population, and surgical treatment is the conventional treatment for low‐grade glioma (LGG). Second, glioblastoma has been identified as an almost incurable form of cancer in response to surgical treatment [5]. Notwithstanding advances in glioma diagnosis, targeted therapy and surgery, and the introduction of criteria such as radiotherapy or chemotherapy, patient prognosis remains poor [6, 7]. Therefore, comprehensive studies are urgently required to fully understand the molecular mechanisms that lead to glioma development, progression, and prognosis.

Neutrophils (NEU) represent the most prevalent innate immune cells and play a pivotal role in innate immunity, including the elimination of bacteria, fungi, parasites, and viruses in infectious diseases [8]. Numerous studies have indicated that neutrophils have an essential function in cancer, encompassing processes such as cancer origin, development, cell growth and proliferation, and metastatic spread at various stages [9]. Previous studies have confirmed that neutrophil counts are crucial in glioblastoma chemotherapeutic response and prognosis for survival [10]. The neutrophil‐to‐lymphocyte ratio (NLR) has been identified as a positive prognostic factor in glioma, with a correlation between NLR and survival in glioma patients [11]. Upon activation, neutrophils may undergo a specific form of programmed cell death, known as NETosis, which results in the formation of neutrophil extracellular traps (NETs). NETs are structures in which material within the nucleus is released to the outside of the cell [12]. Neutrophils have been demonstrated to contribute to tumorigenesis, development, and progression, as well as treatment resistance. This is achieved through a number of mechanisms, including the enhancement of tumor cell proliferation and motility, the promotion of angiogenesis, the release of NETs, the establishment of pre‐metastatic ecological niches, and the mediation of immunosuppression. This apparent contradiction in neutrophil behavior appears to be closely related to their capacity to adapt to different cancer microenvironments [13]. The formation of NETs is one of the critical factors for neutrophils to realize functions [14]. The NETs are web‐like structures rich in chromatin and granule proteins that can cause cell death instead of apoptosis or necrosis [15]. The NETs are related to inflammatory conditions caused by the secretion of inflammatory factors and participate in various diseases, such as rheumatoid arthritis [16], type 2 diabetes [17], and thrombogenesis [18]. More and more research is focusing on the function of NETs in cancer progress. Martins‐Cardoso et al. [19] have confirmed that NETs can promote tumor growth and metastasis in human breast cancer. Elevated levels of NETs in colorectal cancer have been associated with worse clinical outcomes [20]. Studies have shown that NETs interact with hypercoagulability in glioma patients. Therefore, targeting strategies against NETs may provide new directions for the prevention and treatment of thrombotic complications in glioma patients [21]. Additionally, elevated levels of NETs were observed in Grade IV glioma tissues, in which NETs are involved in the proliferation and invasion of glioma cells by activating signaling pathways [22]. Nonetheless, an in‐depth exploration of the specific mechanisms of action of NETs‐related genes (NETs‐RGs) in glioma development and how these findings can be used to develop more effective therapeutic strategies for gliomas remains an urgent need for current research.

This study revolves around NETs‐RGs, which were analyzed and identified in gliomas using the Weighted Gene Co‐Expression Network Analysis (WGCNA) method. Through a series of bioinformatics methods, the potential molecular mechanisms of NETs‐RGs as biomarkers for gliomas were explored in depth and further validated at the cellular and animal levels, aiming to provide new prognostic assessment tools and therapeutic targets for glioma patients, and thus to improve the therapeutic efficacy and prognosis of patients.

## 2 Materials and Methods

### 2.1 Data Sources

The GSE16011 data set downloaded from the Gene Expression Omnibus (GEO) (https://www.ncbi.nlm.nih.gov/geo/) database includes 276 glioma patients and 8 normal samples for differential gene identification (Supporting Information S1 and S2: Tables 1 and 2). The gene expression matrix and clinical data of glioma patients downloaded from The Cancer Genome Atlas Program (TCGA) (https://gdc.cancer.gov/) database, including LGG and glioblastoma, were used for prognostic modeling (Supporting Information S3 and S4: Tables 3 and 4). The clinical information (Supporting Information S5 and S6: Tables 5 and 6) downloaded from the cbioportal website for excluding the IDH mutation in glioblastoma is astrocytoma. These data sets were used as the training sets. This study utilized the gene expression matrices and clinical data from 325 glioma patients in the CGGA data set (http://www.cgga.org.cn/) as an external validation set, excluding astrocytomas through clinical information screening, to validate the constructed model (Supporting Information S7 and S8: Tables 7 and 8). Because of the challenges in obtaining high‐quality, complete clinical and molecular data on Grade 4 cases, the focus of this study was to determine the molecular characteristics of Grades 2 and 3 gliomas. The NETs‐RGs were obtained from previous studies [23], which identified 137 NETs‐RGs associated with the formation and regulation of NETs (Supporting Information S9: Table 9). These genes provided a foundation for us to analyze the potential association between NETs‐RGs and gliomas.

### 2.2 Differential Expression Genes Analysis

To identify genes with significant differences in expression levels among different samples, in the GSE16011 data set, the limma [24] package in R was used to analyze the differential expression of the sample expression matrix (DEGs1), adj.*p*. val < 0.05 and |log_2_FC| > 1 as the screening criteria. The ggplot2 [25] package in R was used to map the volcano plot and heat map.

### 2.3 Weighted Gene Co‐Expression Network Analysis (WGCNA)

To find co‐expressed gene modules and to explore the association between gene networks and traits of interest, the WGCNA [26] package in R was used for the WGCNA weighted co‐expression network analysis of glioma samples in TCGA [26]. First, using the ssGSEA algorithm from the GSVA package (v 1.42.0) [27], we calculated NETs‐RGs ssGSEA scores for all samples. Correlation coefficients and *p* values between WGCNA modules and NETs‐RGs scores were computed through relevance analysis, and visualized as a heat map to single out the module most tightly correlated with the ssGSEA score. Venn diagrams were generated using the VennDiagram package (v 1.7.1) [28] in R for further data comparison and visualization.

### 2.4 GO Enrichment Analysis

In order to investigate the biological functions and signaling pathways involved in candidate genes, the GOplot [29] package in R was utilized for Gene Ontology (GO) [30] enrichment analyses of differentially expressed candidate genes, with *p* adjust < 0.05 as the screening criteria.

### 2.5 Establishment Risk Model

The survival [31] package in R was used to perform a univariate proportional hazards (PH) model (Cox regression model [32]) analysis (HR ≠ 1, *p* < 0.05) and the PH assumption test (*p* > 0.05) to identify candidate prognostic‐related genes significantly associated with the prognosis of glioma patients. Among these genes, those that met the criteria for univariate Cox regression analysis but violated the PH assumption were analyzed using an extended Cox model incorporating a time interaction term to obtain more accurate effect estimates. Specifically, a time‐varying coefficient Cox model was constructed by introducing a time interaction term (gene × log(*t*)) [33, 34, 35]. The forestplot [36] package in R for mapping the forest maps. Subsequently, the glmnet [37] package in R for performing Least Absolute Shrinkage and Selection Operator (LASSO) regression analysis and establishing the risk model, the best model was created when lambda was smallest (lambda.min). Genes with non‐zero coefficients in the model were identified as prognostic‐related genes. The LASSO model employed 10‐fold cross‐validation to avoid model overfitting and enhance the model's generalization ability. The risk score formula:

$\text{Risk score}=\sum_{i=1}^{n}\mathrm{coef}\left({\mathrm{gene}}_{i}\right)*\mathrm{expr}({\mathrm{gene}}_{i}).$

Coef represented the risk coefficient of each gene, and expr represented the expression level of each gene.

The BCR survival curve was mapped using the survminer [31] package in R, and the survivalROC [38] package was used to plot the receiver operating characteristic (ROC) curve. The log‐rank testing for performing Kaplan–Meier survival curve analysis. The rms [39] package was used to map the nomogram of predicting TCGA‐Glioma patients' death rate.

### 2.6 Gene Set Enrichment Analysis (GSEA)

GSEA analysis allows the identification of pathways and biological processes that are differentially regulated between groups, thereby deepening the understanding of the mechanisms behind the observed changes in gene expression. GSEA enrichment analysis was performed (Hallmark.symbols.gmt) as the reference gene set from MSigDB (https://www.gsea-msigdb.org/gsea/msigdb/) database, gene sets with FDR < 0.05 were considered significantly enriched. The enrichplot [40] package was used to visualize the results.

### 2.7 Immune Infiltration and Drug Sensitivity Analysis

Differences between samples from high‐ and low‐risk glioma groups and the immune microenvironment explored by immune infiltration analysis. The immune infiltration cell analysis used the CIBERSORT algorithm for sample gene expression data in the TCGA‐Glioma set and evaluated relative abundance in 22 immune infiltration cells of all samples. The Wilcoxon tested the immune cell infiltration differential between high‐ and low‐risk groups. The estimation of stromal and immune cells in malignant tumors using expression data algorithm used the estimate [41] package to measure. Wilcoxon tested the expression levels.

Association of prognosis‐related genes with response to chemotherapy assessed by drug sensitivity analysis. In the TCGA‐Glioma set, pRRophetic [42] package was used to measure 138 common chemotherapy and molecular‐targeted drugs with IC_50_ values of all patients. The IC_50_ is the semi‐inhibitory concentration, which is usually used to measure the toxicity of a drug to cells or the degree of cellular tolerance to a drug, and the lower the IC_50_, the stronger the performance of the drug. The correlation analysis between related candidate genes and drugs and screening the drugs of Spearman > 0.9. Wilcoxon tested the expression levels of common chemotherapy drugs with IC_50_.

### 2.8 Clinical Samples

In 2024 years, a total of 30 glioma patients' tissue samples, and normal brain tissue samples were collected. The study was approved by the Ethics Committee of Kunming Medical University, and all patients signed an informed consent form. Tissue samples were preserved by liquid nitrogen or fixed in 4% paraformaldehyde and used for subsequent experiments.

### 2.9 Cell Culture

Human astrocytes (NHA, BFN60808805) were obtained through the Shanghai Cell Bank, China, and human glioma cell lines H4 (CL‐0087), A172 (CL‐0012), LN229 (CL‐0578), U87 (CL‐0238), and U251 (CL‐0237) were purchased from Wuhan Prosperity Life Sciences Co. The purchased cells were cultured by DMEM cell culture medium (Sigma‐Aldrich, MO, USA) containing 10% fetal bovine serum (FBS; A5669701, ThermoFisher Scientific), 1% double antibody (Sigma‐Aldrich). They were incubated and cultured in a cellular thermostat (37°C) incubator containing 5% CO_2_, and passaged when the cells grew 80%–90% of the dish.

### 2.10 Cell Transfection

The cells were cultured overnight in a 24‐well plate. When the cell density reached about 60%–70%, si‐NC and si‐NFIL3 were transfected into the cells according to the instructions of the Lipofectamine 3000 reagent (Invitrogen, Grand Island, NY, USA). The cells were cultured at 37°C and 5% CO_2_ incubator for 48 h, and the transfection efficiency was detected.

### 2.11 RT‐qPCR

The total RNA of the tissue sample and cells were extracted with TRIzol reagent (Invitrogen, 15596026) and reverse transcribed into single‐strand complementary DNA (cDNA) by One Step Prime Script miRNA cDNA Synthesis Kit (Takara, Kyoto, Japan). Using SYBR Green PCR Master Mix (Life Technologies, CA, USA), follow the manufacturer's procedures for RT‐qPCR. The sequence of RT‐qPCR primers is shown in Table 1. Taking GAPDH as the internal reference, the value was calculated by the 2^−ΔΔCt^ method.

**Table 1:** PCR primer sequence.

Genes	Primer	Sequence (5′–3′)
ALDH1A3	Forward	5′‐TTCAACTCGGGAGCAAAT‐3′
	Reverse	5′‐CATGCCTGGTGAAGCACA‐3′
CHCHD10	Forward	5′‐ATGGCTCAGATGGCGACC‐3′
	Reverse	5′‐TCAGGGCAGGGAGCTCAG‐3′
NFIL3	Forward	5′‐AAGCAAGAGCCGATGGAA‐3′
	Reverse	5′‐TGTGGCAAGGCAGAGGAA‐3′
GAPDH	Forward	5′‐TGACCACAGTCCATGCCATCAC‐3′
	Reverse	5′‐CGCCTGCTTCACCACCTTCTT‐3′

### 2.12 Western Blot

Total proteins of tissue sample and cell were extracted with RIPA buffer (Sigma‐Aldrich, USA) containing 1% protease inhibitor and phosphatase inhibitor, separated with 10% SDS‐PAGE gel, and transferred to polyvinylidene fluoride membrane (Millipore, MA, USA). At room temperature, the film was sealed with 5% skim milk for 2 h, the first antibody was added and spent the night at 4°C, and then the second antibody with HRP conjugated (1:2000, ab205718, Abcam, UK) was incubated at room temperature for 1 h. ECL chemiluminescence solution development (BD Biosciences) was used for exposure and observation, and ImageJ was used for protein band analysis. GAPDH (1:1000, ab8245, Abcam, UK) or β‐actin antibody (1:1000, ab8226, Abcam, UK) was used as a control. The primary antibodies purchased from Abcam (UK) following: anti‐ALDH1A3 (1:1000, ab129815), anti‐CHCHD10 (1:500, ab308511), anti‐NFIL3 (1:1000, ab230090), anti‐Cleaved‐caspase‐3 (1:500, ab32042), anti‐Bax (1:1000, ab32503), and anti‐Bcl‐2 (1:2000, ab182858) purchased from Cell Signaling, USA.

### 2.13 CCK‐8 Detection

The CCK‐8 test kit (Biyuntian, Beijing, China) was used to detect cell viability. Each group of cells was added to a 96‐well plate at a concentration of 3 × 10^3^ cells per. Subsequently, 10 μL of CCK‐8 solution was introduced and incubated at 37°C for 1–3 h. An enzyme labeling instrument was utilized to measure the absorbance values at 450 nm.

### 2.14 Cell Migration, Invasion, and Colony Formation Assay

Cells were seeded in 6‐well plates, one scratch per well was made with a sterile gun head, and PBS was used for cleaning the cell debris and taken at 0 and 24 h. ImageJ was used to detect the cell migration.

Cells were adjusted to 5 × 10^4^ without FBS DMEM solution and joined the Transwell (Corning, USA) upper chamber precoated with Matrigel (Corning). Then, the lower one joined in with FBS DMEM solution after culture for 24 h, stained with crystal violet, and observed under an inverted microscope (CKX53, OLYMPUS, Japan). Finally, ImageJ was used to detect the cell invasion rate.

Cells were adjusted to 1000/well seeded in 6‐well plates and cultured for 2 weeks. Then, 4% paraformaldehyde was used to fix, and crystal violet was used to stain cells. A light microscope and ImageJ were used to analyze the number of clones.

### 2.15 Flow Cytometry

After the cells were collected, they were rinsed twice with PBS and resuscitated with 200 μL PBS. The rate of programmed cell death was measured using the Annexin‐V‐FITC/PI Apoptosis Kit from Absin, China. In total, 5 μL of Annexin V‐FITC and 5 μL of PI were added to each well according to the guidelines provided by the manufacturer, and the cells were incubated in a darkened chamber for 15 min before apoptosis was identified using a FACScan flow cytometer.

### 2.16 Immunohistochemical

The tissue samples used paraffin slice sections stained with immunohistochemistry (IHC) by anti‐ALDH1A3, anti‐CHCHD10, and anti‐NFIL3 and joined in HRP antibody and DAB chromogenic agent to immune testing. The results were photographed under a microscope (400857, Nikon, Japan).

### 2.17 Xenograft Mouse Experiments

BALB/c nude mice (4–6 weeks, 20 ± 2 g) were purchased from SPF (Beijing) Biotechnology Co. All mice were raised in a regulated environment according to specific pathogen free (SPF) standards. Briefly, after transfection, 100 μL (1 × 10^6^ cells) of LN229 or U87 cells were injected into the right side of each nude mouse to establish a subcutaneous tumor model (*n* = 6). The Control group was injected with nontransfected LN229 or U87 cells, and after 1 week of injection, the subcutaneous tumor was measured every 3 days with a vernier caliper for a total of 21 days. Each mouse was necropsied at the end of 4 weeks postinjection and tumors were extracted for evaluation.

### 2.18 Statistical Analysis

All experimental data in this study are expressed as mean ± SD. Before conducting *t*‐tests and ANOVAs, we verified whether the data met the fundamental assumptions for parametric tests. All continuous variables were assessed for normal distribution using the Shapiro–Wilk test. Homogeneity of variance was evaluated via Levene's test. For comparisons between two independent samples, Student's *t*‐test was applied when data met the assumption of equal variances; Welch's *t*‐test was used for correcting unequal variances. For multiple group comparisons, one‐way ANOVA was performed if homogeneity of variance was satisfied, followed by Tukey's honest significant difference test for post hoc multiple comparisons. When variance was unequal, Brown–Forsythe ANOVA was used, followed by Games–Howell test for post hoc analysis. All statistical analyses were performed using GraphPad Prism 8 software. *p* < 0.05 was considered statistically significant.

## 3 Results

### 3.1 The Results of Differential Expression Analysis in GSE16011

**Figure 1:**
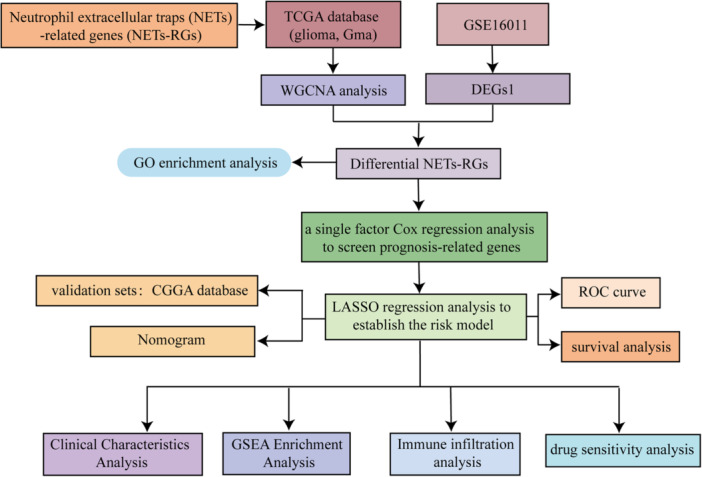
Bioinformatics analysis flowchart.

**Figure 2:**
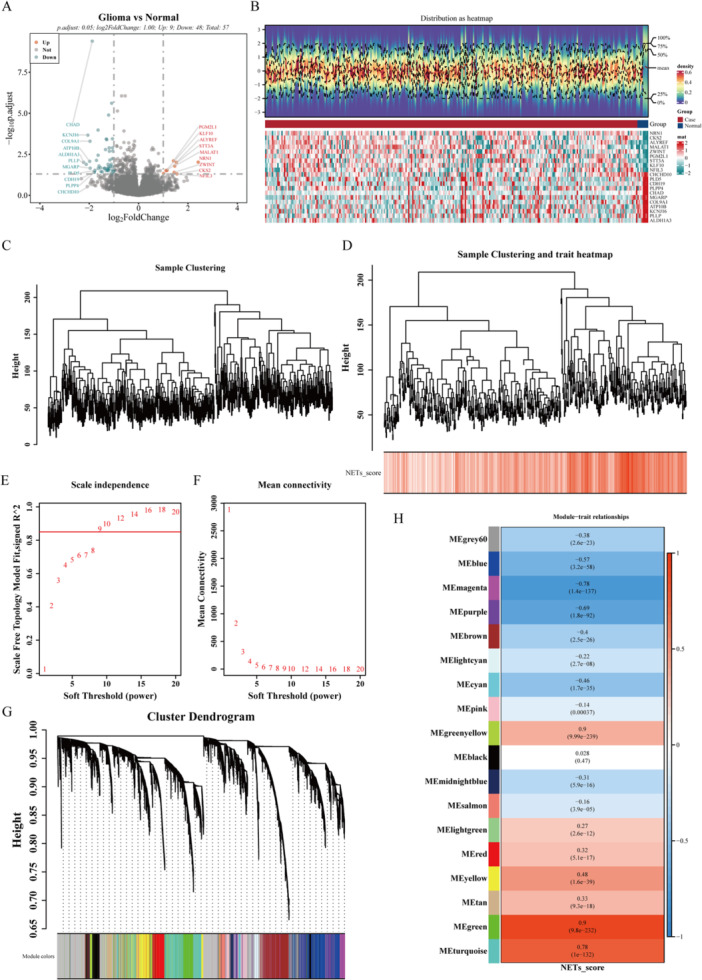
The results of differential expression analysis in GSE16011. (A) Volcano plot of differential expression genes in GSE16011. Red dots were upregulated genes, green dots were downregulated genes, and gray dots were no differentiated genes. (B) Heat map of differential expression genes in GSE16011. Red dots represented high expression, green dots represented low expression. (C) Sample‐level clustering. Each branch in the clustering tree represented a sample, the red line represented the cut line, and the vertical coordinate represented the sample expression Euclidean distance. (D) Sample‐level clustering after the introduction of sample traits. (E and F) Soft threshold sievescreen for soft thresholds. Determining the optimal soft threshold is mainly referred to the E plot, that is, the scale‐free fit index (scale‐free fit index, *y*‐axis) in the case of different soft thresholds (*x*‐axis). Index (*y*‐axis) for different soft thresholds (*x*‐axis). The red line indicates the selected value of scale‐free fit index. From the left figure, when the scale‐free fit index is 0.85, it is consistent with the minimum scale‐free fit index for constructing the scale‐free network. The right figure shows the network connectivity under different soft thresholds. (G) Identification of co‐expression modules: The top part was gene‐level clustering, and the bottom part was gene modules, corresponding to the top and bottom. (H) Heat map of modules and correlation, the left color represented modules, the right color represented correlation, red was positive, and blue was negative.

The flowchart is presented in Figure 1. To explore the key genes in glioma, we analyzed the differential expression genes between Glioma and Normal samples in GSE16011 (Supporting Information S10: Table 10), and obtained 57 differential expression genes (DEGs1, Supporting Information S11 and S12: Tables 11 and 12), including 9 upregulated genes and 48 downregulated genes (Supporting Information S13: Table 13), as shown in Figure 2A,B. To clarify the relationship between NETs‐RGs and glioma, we further conducted the WGCNA analysis. The results demonstrated that no significant outlier samples were present in the data set, eliminating the necessity for their exclusion (Figure 2C,D), the NETs score was shown in Supporting Information S14: Table 14. As shown in Figure 2E, *R* ^2^ = 0.85 was set to screen for soft thresholds above the red cut line. As shown in Figure 2F, the soft thresholds with a connectivity trend of 0 were screened, and finally, the soft threshold *β* = 9 was selected. WGCNA analysis obtained 18 co‐expressed modules, shown in Figure 2G (excluding the gray modules that contained unclassified genes). Based on NETs‐RGs, we measured the ssGSEA score of all samples and used relevance analysis to calculate the correlation coefficients and p‐values for the WGCNA module and NETs‐RGs score and the heat map shown in Figure 2H. The green and turquoise modules were selected on the basis of the highest positive correlation with phenotype, with the green module containing 931 genes and the turquoise module containing 2046 genes (Supporting Information S15: Table 15).

### 3.2 Identification of Candidate Genes

**Figure 3:**
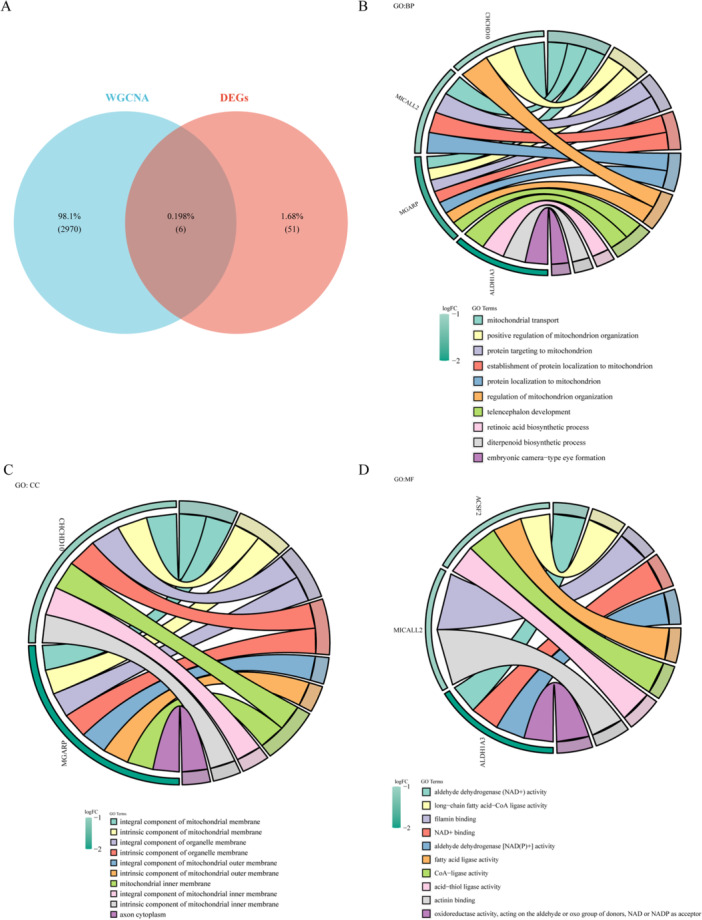
Identification of candidate genes. (A) Venn diagram of DEGs and WGCNA modular genes. GO function analysis of genes, including BP terms (B), CC terms (C), and MF terms (D), only showed the Top 10.

Furthermore, the intersection between DEGs1 and above the green and turquoise modules with the highest positive correlation genes was identified. This resulted in the selection of 6 intersection genes (Supporting Information S16: Table 16) as the candidate genes (Figure 3A), including ACSF2, ALDH1A3, CHCHD10, MGARP, MICALL2, and NFIL3. The results of function enrichment analysis are shown in Figure 3B–D. It was enriched to 74 GO biological functions (Supporting Information S17: Table 17), including 43 BP terms (mitochondrial transport, positive regulation of mitochondrion organization, protein targeting to mitochondrion, establishment of protein localization to mitochondrion, and protein localization to mitochondrion), 17 CC terms (integral component of mitochondrial membrane, intrinsic component of mitochondrial membrane, integral component of organelle membrane, intrinsic component of organelle membrane, and integral component of mitochondrial outer membrane), and 14 MF terms (aldehyde dehydrogenase (NAD+) activity, long‐chain fatty acid‐CoA ligase activity, filamin binding, NAD+ binding and aldehyde dehydrogenase [NAD(P)+] activity).

### 3.3 The Results of Establishing and Validating the Risk Model

**Figure 4:**
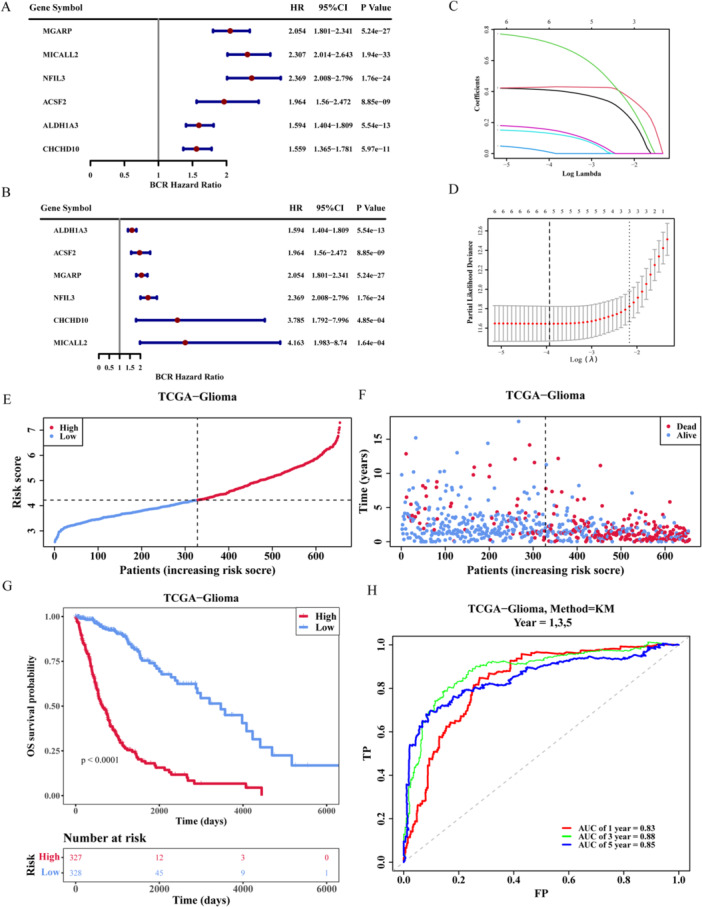
The results of establishing the risk model. (A) The forest map of univariate Cox regression analysis. (B) Forest plot of time‐varying coefficient Cox model for six genes in glioma patients. (C and D) The LASSO analysis was utilized to identify genes that are related to prognosis. (E) The risk curve of glioma patients high‐ and low‐risk in training sets. (F) Scatter plot of glioma patients high‐ and low‐risk in training sets. (G) Survival curve of glioma patients high‐ and low‐risk in training sets. (H) ROC curve of glioma patients 1, 3, and 5 years in training sets.

The relationship between candidate genes and glioma prognosis was evaluated using a Cox regression analysis. The univariate Cox regression analysis yielded six survival‐related genes, as illustrated in Figure 4A, respectively, MGARP, MICALL2, NFIL3, ACSF2, ALDH1A3 and CHCHD10 (Supporting Information S18 and S19: Tables 18 and 19). The PH test showed that the *p* values of MICALL2 and CHCHD10 were < 0.05, indicating that the prognostic associations of these two genes with glioma patients changed over time (Supporting Information S20: Table 20). Therefore, a time‐varying coefficient Cox model for the six genes was further constructed (Figure 4B). Furthermore, a LASSO regression analysis was employed to construct a risk model comprising the six aforementioned genes (Supporting Information S21: Table 21). This model is illustrated in Figure 4C,D and was derived when the lambda_min_ = 0.01957122, corresponding to six genes. Consequently, a total of six genes were identified as prognosis‐related genes (ACSF2, ALDH1A3, CHCHD10, MGARP, MICALL2, and NFIL3). The results of the risk score are shown in Figure 4E,F; the number of deaths increased while the risk score rose (Supporting Information S22: Table 22). The BCR survival curve shown in Figure 4G indicates that the patients in the high‐risk group had a lower survival rate. The ROC curve shown in Figure 4H indicates that the AUC > 0.6, so our risk model had an excellent predicted performance.

**Figure 5:**
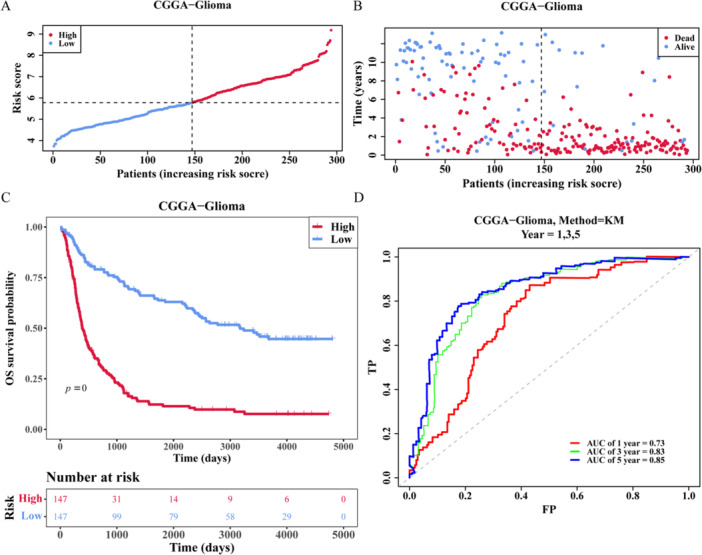
The results of validating the risk model. (A) The risk curve of glioma patients high‐ and low‐risk in CGGA. (B) Scatter plot of glioma patients high‐ and low‐risk in CGGA. (C) Survival curve of glioma patients high‐ and low‐risk in CGGA. (D) ROC curve of glioma patients 1, 3, and 5 years in CGGA.

Subsequently, the risk model was validated using the CGGA validation sets (Supporting Information S23: Table 23). The results showed that the number of deaths increased while the risk score rose (Figure 5A,B). The prognosis model demonstrated efficacy in predicting the survival status of high‐ and low‐risk groups in validation sets (Figure 5C), with an AUC > 0.6 in both of the 1–5 years nodes on the ROC curve (Figure 5D). These results indicated that the prognosis model could be a good predictor of a patient's glioma risk.

### 3.4 The Results of Independent Prognostic Analysis, the Nomogram Established and the GSEA Analysis

**Figure 6:**
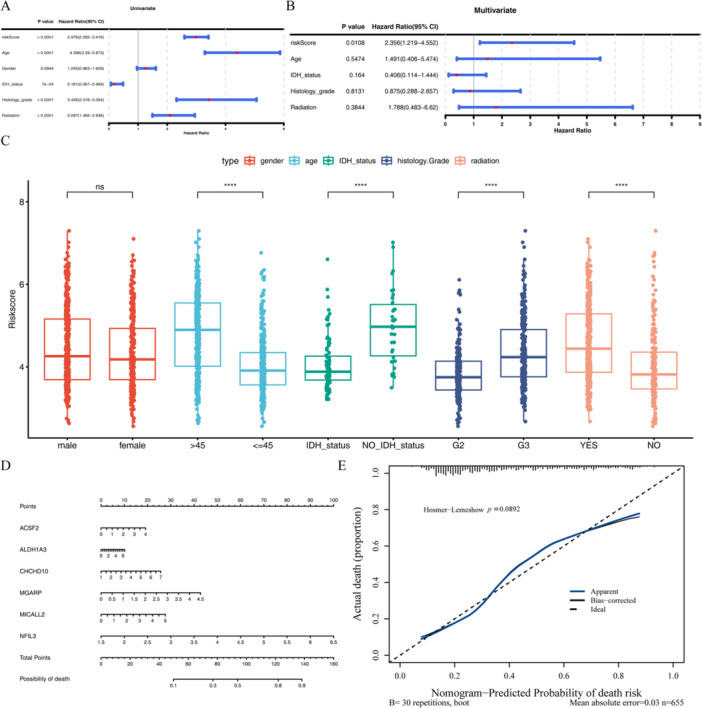
The results of the independent prognostic analysis and the nomogram established. (A) The univariate regression analysis of clinical characteristics and risk scores in glioma. (B) The multifactorial regression analysis of clinical characteristics and risk scores in glioma. (C) The risk score in different clinical characteristics subgroups. (D) The nomogram of predicting TCGA‐Glioma patients' death rate. (E) Calibration curve of gene nomogram. ^ns^ *p* > 0.05, ****p < 0.0001.

**Figure 7:**
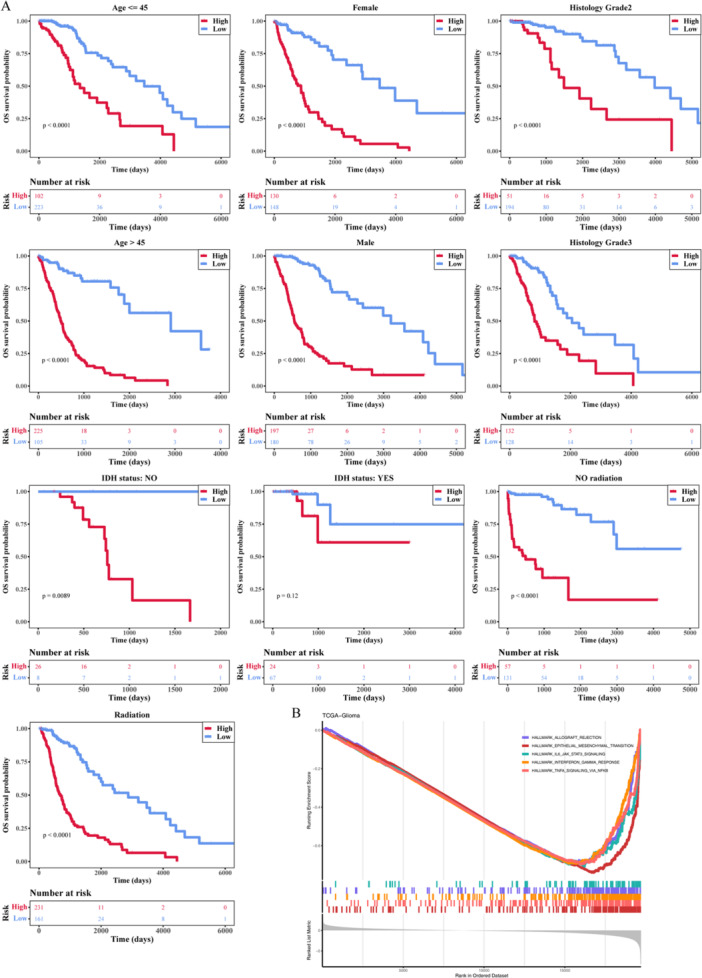
The results of Kaplan–Meier survival curve analysis and GSEA analysis. (A) KM survival curve of clinical characteristics between the two subtypes. (B) The results of the GSEA analysis.

To further research the prognostic risk model and clinicopathological characteristics, we choose factors such as age, Gender, IDH mutant, Histology grade, and radiation with risk score added to the risk model to perform univariate and multivariate Cox independent prognostic analysis (Supporting Information S24–S27: Tables [Link], [Link], [Link], [Link]). Showed in Figure 6A,B that both of *p* value < 0.05 in riskScore, age, IDH mutant, Histology grade, and radiation. The results showed that the *p* values of the risk scores were < 0.05 in both univariate and multivariate Cox independent prognostic analyses. And four clinicopathological characteristics risk scores had significant differences with subgroups including age, IDH mutant, Histology grade, and radiation (Figure 6C). The Kaplan–Meier survival curve analysis results showed in Figure 7A that only the *p* > 0.05 (*p* = 0.12) of IDH mutant was in five clinicopathological characteristics. The nomogram of predicting TCGA‐Glioma patients' death rate is shown in Figure 6D,E, illustrating that the nomogram had a good prognosis value (*p* > 0.05). Second, the results of GSEA analysis (Supporting Information S28 and S29: Tables 28 and 29) enriched in such as EPITHELIAL_MESENCHYMAL_TRANSITION, TNFA_SIGNALING_VIA_NFKB, INTERFERON_GAMMA_RESPONSE, ALLOGRAFT_REJECTION, IL6_JAK_STAT3_SIGNALING (Figure 7B).

### 3.5 The Results of Immune Infiltration Analysis

**Figure 8:**
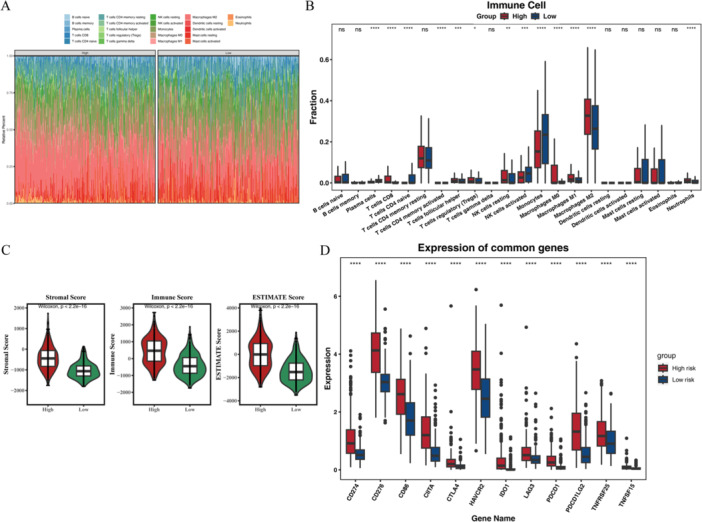
The results of immune infiltration analysis. (A) The ratio of immune cells in high‐ and low‐risk groups. (B) The differences in immune cells between the high‐ and low‐risk groups. (C) Stromal, Immune, and ESTIMATE scores between high‐ and low‐risk groups. (D) The differences of 12 immune checkpoint molecules between the high‐ and low‐risk groups. ^ns^ *p* > 0.05, *p < 0.05, **p < 0.01, ***p < 0.001, ****p < 0.0001.

To further explore the difference between glioma high‐ and low‐risk and immune microenvironment, Figure 8A shows the ratio of high‐ and low‐risk group samples in 22 immune infiltration cells (Supporting Information S30: Table 30). A total of 13 immune cells had significant differences between high‐ and low‐risk group samples (Figure 8B). To evaluate the value of immune and stromal cells in the tumor microenvironment, the ESTIMATE results showed the Stromal, Immune, and ESTIMATE scores between high‐ and low‐risk groups (Figure 8C). Finally, we used Wilcoxon to detect the expression levels of 12 immune checkpoint molecules (Supporting Information S31: Table 31) between the high‐ and low‐risk group, found that the 12 immune checkpoint molecules both had significant differences (Figure 8D), including PDCD1, CD274, PDCD1LG2, CTLA4, IDO1, CD276, LAG3, HAVCR2, TNFRSF25, TNFSF15, CD86, and CIITA.

### 3.6 Drug Sensitivity Analysis and the Expression Levels Validation

**Figure 9:**
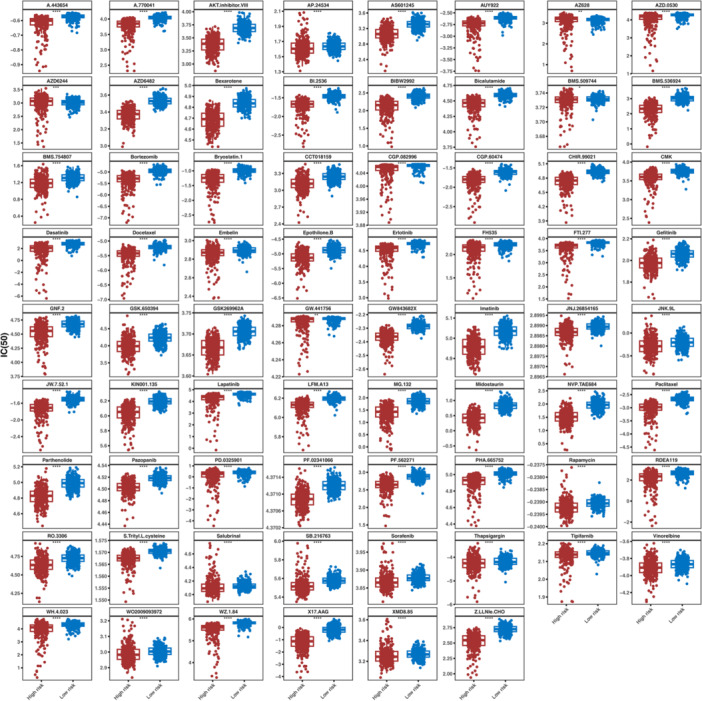
The IC_50_ of common chemotherapy drugs between high‐ and low‐risk groups (low expression).

**Figure 10:**
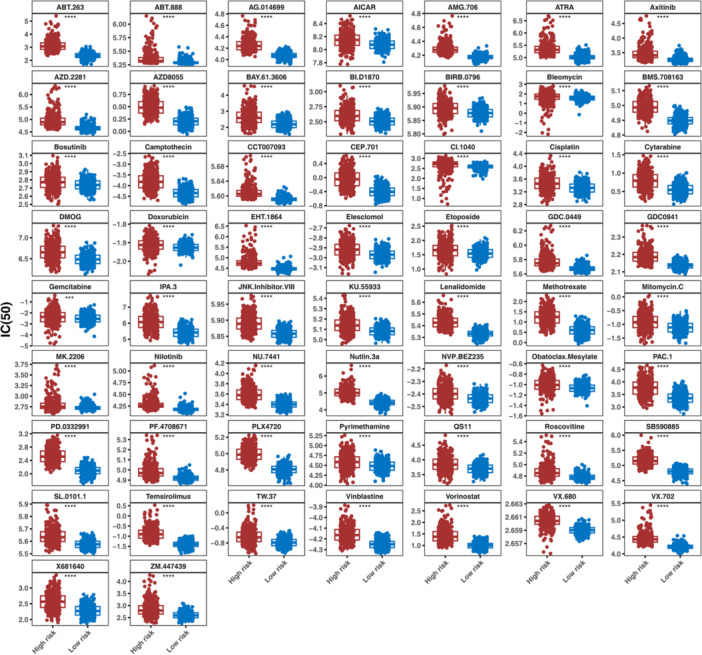
The IC_50_ of common chemotherapy drugs between high‐ and low‐risk groups (high expression).

**Figure 11:**
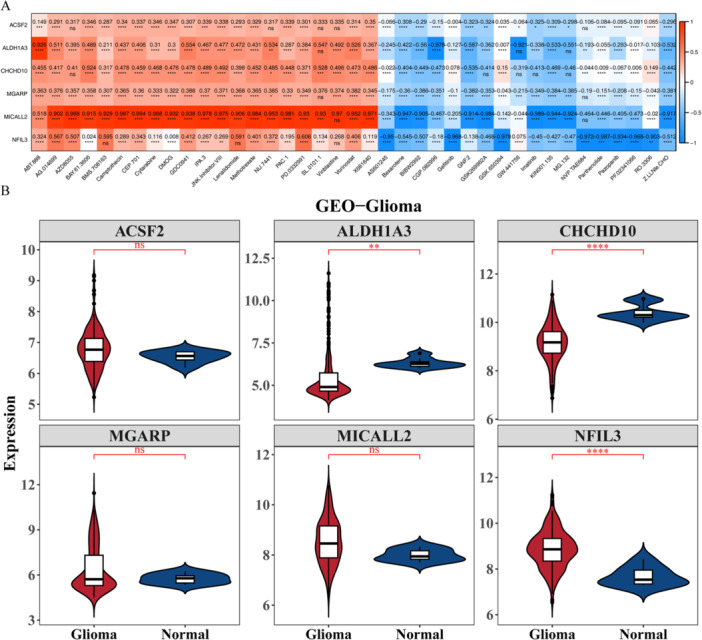
Drug sensitivity analysis and the expression levels validation. (A) The correlation heat map of prognosis‐related genes with drugs. (B) The expression levels of prognosis‐related genes in GSE16011 sets. ^ns^ *p* > 0.05, **p < 0.01, ****p < 0.0001.

We aimed to explore if genetic changes tied to NETs scores directly impact drug sensitivity in glioma through a dedicated drug sensitivity experiment. To further estimate the relationship between prognosis‐related genes and chemotherapy response, we measured the IC_50_ of 138 common chemotherapy and molecular targeted drugs in all patients (Supporting Information S32: Table 32) and inspected the expression differences of common chemotherapy drugs between high‐ and low‐risk groups, showed in Figures 9 and 10, a total of predicted 128 differences chemotherapy drugs, including 70 chemotherapy drugs had low expression, 58 chemotherapy drugs had high expression in high‐risk group. We screened out the drugs with absolute correlation value > 0.9 with prognosis‐related genes, and the correlation analysis results found that MICALL2 had a significant correlation with multiple drugs (Figure 11A and Supporting Information S33: Table 33). Next, in GSE16011 sets, analyzed the expression levels of six prognosis‐related genes between Glioma and Normal samples, shown in Figure 11B, showed significant differences of ALDH1A3, CHCHD10, and NFIL3.

### 3.7 Validation of the Expression Levels of Key Genes

**Figure 12:**
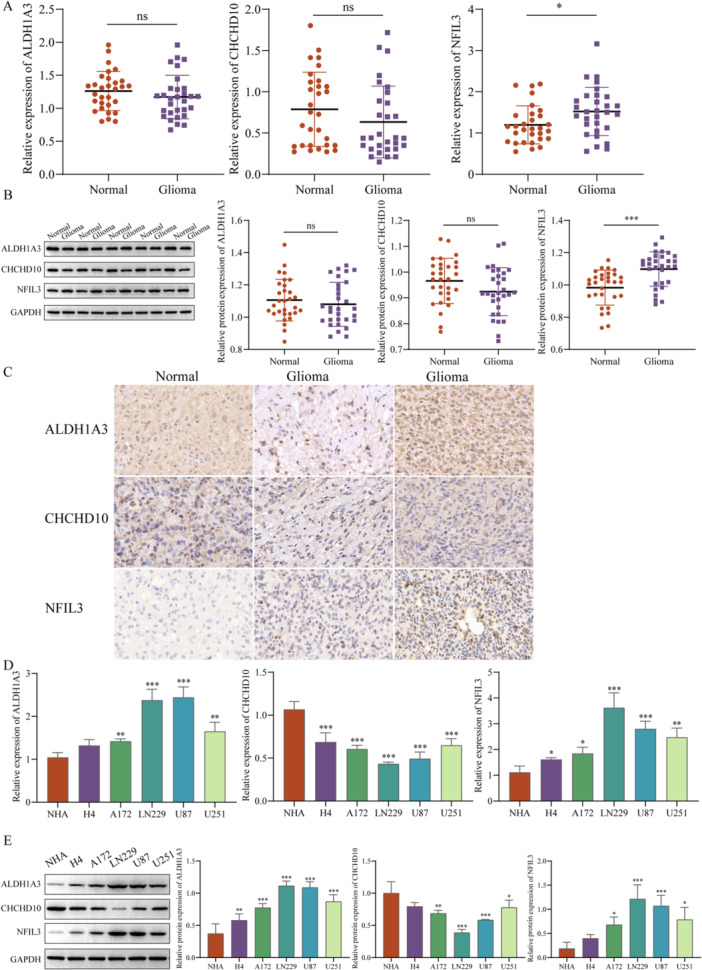
Identification of key genes. The RT‐qPCR (A), western blot (B), and IHC (C) detected the expression levels of key genes in glioma patients’ tissue samples. Scale bar = 20 μm. The RT‐qPCR (D) and western blot (E) detected the expression levels of key genes in glioma cells. ^ns^ *p* > 0.05, *p < 0.05, **p < 0.01, ***p < 0.001.

To further understand the mechanism of action of key genes, we collected glioma patients' tissue samples, to further verify the expression levels of key genes, the RT‐qPCR (Figure 12A), western blot (Figure 12B), and IHC (Figure 12C) both showed a same result of the expression level of ALDH1A3 both high expression and low expression, and CHCHD10 had a no significant difference in expression, while NFIL3 was significant high expression levels. Moreover, ALDH1A3 had high expression levels in the glioma cells, NFIL3 had a significantly high expression, especially in LN229 and U87 cells, while CHCHD10 had low expression levels in the glioma cells (Figure 12D,E). Therefore, we will further explore the mechanism of action of NFIL3 in glioma in subsequent experiments.

### 3.8 NFIL3 Affects the Malignant Progression of Glioma Cells

**Figure 13:**
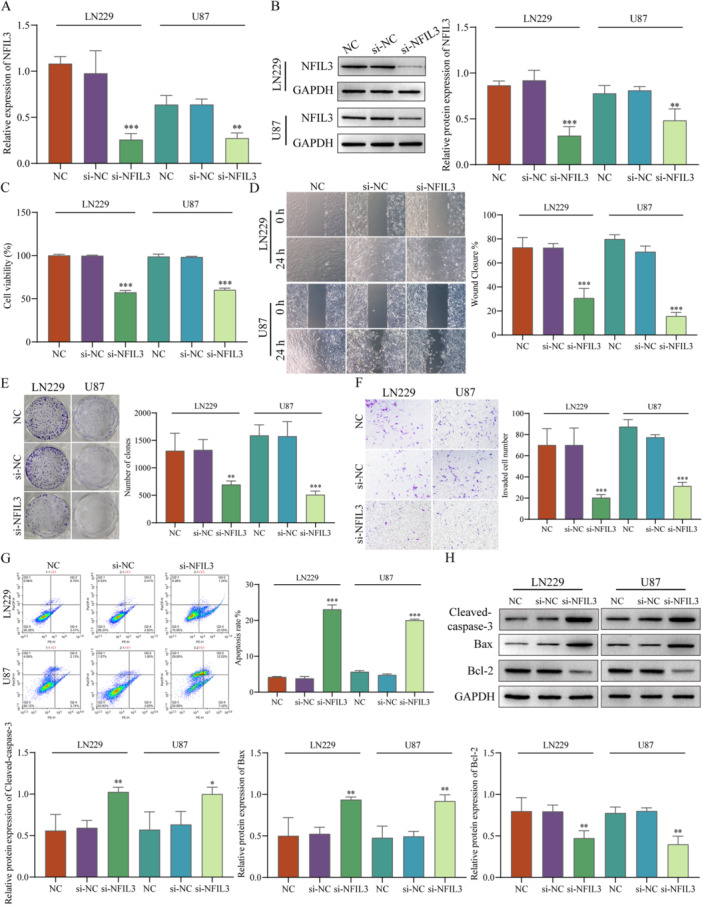
NFIL3 affects the malignant progression of glioma cells. RT‐qPCR (A) and western blot (B) were used to evaluate transfection efficiency. (C) Cell viability was detected by CCK‐8. Cell scratch assay (D), clone assay (E), and Transwell assay (F) were used to evaluate cell migration and invasive ability. (G) Flow cytometry detected apoptosis. (H) Western blot detected the protein expression of Cleaved‐caspase‐3, Bax and Bcl‐2. *p < 0.05, **p < 0.01, ***p < 0.001.

RT‐qPCR and western blot were used to evaluate transfection efficiency, after transfecting si‐NC and si‐NFIL3 into LN229 and U87 cells, the results showed that the expression levels of NFIL3 were significantly decreased in the si‐NFIL3 group, while compared with NC group, the si‐NC group no significant difference (Figure 13A,B), transfection was successful. After the knockdown of NFIL3, the cell viability was significantly reduced in LN229 and U87 cells (Figure 13C). The results of the cell scratch assay (Figure 13D), clone assay (Figure 13E), and Transwell assay (Figure 13F) showed that knockdown of NFIL3 inhibited LN229 and U87 cells migration and invasive ability. Moreover, flow cytometry (Figure 13G) and western blot (Figure 13H) found that knockdown of NFIL3 promoted apoptosis, upregulated the protein expression of Cleaved‐caspase‐3 and Bax, downregulated Bcl‐2. Therefore, these results showed that knockdown of NFIL3 can inhibit LN229 and U87 cell proliferation, migration and invasion ability, and promote apoptosis.

### 3.9 NFIL3 Affects the Growth of Glioma Cells In Vivo

**Figure 14:**
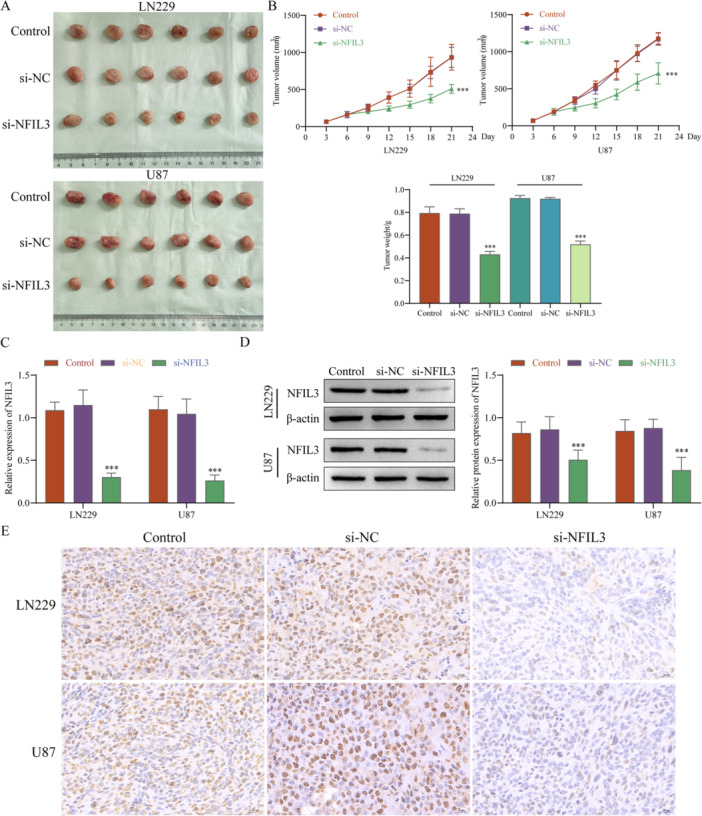
NFIL3 affects the growth of glioma cells in vivo. (A) Tumor growth image. (B) Tumor volume and weight. The RT‐qPCR (C), western blot (D), and IHC (E) detected the expression levels of NFIL3 in tumor tissue. Scale bar = 20 μm. ***p < 0.001.

Xenografting of successfully transfected si‐NC and si‐NFIL3 LN229 and U87 cells into mice revealed that the tumor growth was significantly inhibited after transfecting si‐NFIL3, while there was no significant difference between the NC group and si‐NC group (Figure 14A,B). RT‐qPCR, western blot, and IHC were used to detect the expression level of NFIL3 in tumor tissue, found that the expression levels of NFIL3 were significantly decreased in the si‐NFIL3 group (Figure 14C–E). The above results show that knockdown of NFIL3 can inhibit LN229 and U87 cell growth in vivo.

## 4 Discussion

Gliomas are prevalent brain tumors that have a high degree of invasiveness and resistance to drugs, hence significantly complicating treatment [43]. Finding novel biomarkers and improving the prognosis value for diagnosing and treating glioma is essential. In this study, we conducted a differential expression analysis, WGCNA analysis, univariate and multifactorial Cox analysis, LASSO analysis, survival analysis, independent prognostic analysis, GSEA analysis, immune infiltration analysis, drug sensitivity analysis and expression level validation using the transcriptome data of glioma patients from the GEO database, transcriptome data and clinical data of glioma patients from the TCGA‐Glioma database, and gene expression matrix and clinical data of glioma patients from he CGGA database. Ultimately, 6 prognosis‐related genes of glioma were identified: MGARP, MICALL2, NFIL3, ACSF2, ALDH1A3, and CHCHD10. Based on the differential expression patterns they exhibited in NETs‐RG analysis that were strongly associated with glioma occurrence and progression. We selected these genes as the basis for the glioma risk score. The expression changes of these genes may directly map the biology of glioma cells during the formation and regulation of NETs. By constructing a risk scoring model, we could quantitatively assess the expression levels of these genes in gliomas and their impact on prognosis. WGCNA, GO, and GSEA analyses allowed us to explore the importance of these genes in gliomas, and the results of the analyses revealed the biological pathways, functional networks, and functional enrichment to which they belonged, and validated their association with gliomas.

NETs have complex and significant roles in the progression of various types of tumors [44]. Previous research has reported the formation of NETs in diseases affecting the CNS, and NETs have a high level of high‐grade glioma (HGG) [22]. NETs can enhance thrombogenicity by endothelial cells in glioma patients [21]. Hence, the role of NETs is crucial in the mechanisms involved in the progression of glioma. In this study, we screened the differential expression genes between Glioma and Normal samples and used the WGCNA analysis, which has been widely used to identify biomarkers in various types of cancer [45], to find two modules with the highest positive correlation with NETs‐RGs, obtained six candidate genes: ACSF2, ALDH1A3, CHCHD10, MGARP, MICALL2, and NFIL3. Also, we found candidate genes were related to protein targeting the mitochondrion and mitochondrial membrane. Target mitochondria‐induced cell apoptosis has been confirmed to be an effective anti‐glioma treatment method [46]. Based on the candidate genes to establish the risk model and independent prognostic analysis, finally ensured the 6 genes had a significant correlation with the survival of glioma patients by univariate and multifactorial Cox analysis and LASSO analysis when the risk score was higher, the survival was poorer of patients. Risk scores are widely used in clinical practice to assess a patient's probability of developing a particular disease. Recent studies have shown that polygenic risk scores have a significant advantage over rare single‐gene mutations in identifying people at high risk for a wide range of common diseases, and are able to cover a larger proportion of the population at comparable or higher risk for disease [47]. Notably, our study further revealed that four key clinicopathological characteristics (age, IDH mutation status, histological grading, and radiation exposure) were significantly different from the risk scores of specific subgroups. This finding not only deepens our understanding of disease risk stratification, but also heralds the great potential application of our risk scoring system in the field of precision medicine, which can provide strong support for early warning, development of personalized treatment strategies, and efficacy monitoring of diseases such as glioma.

The immune microenvironment regulates immune responses by releasing cytokines, further inducing cell immune infiltration [48]. The immune cell infiltration in the tumor microenvironment has been verified to affect glioma prognosis [49, 50]. In this study, we observed significant differences in immune infiltrating cells between high‐risk and low‐risk glioma groups, and in particular, 13 immune cells showed significant group differences, findings that reveal the complexity of the immune microenvironment of gliomas and its potential link to disease risk. The IC_50_ values of 138 common chemotherapeutic and molecular‐targeted drugs were analyzed and found expression differences on 128 chemotherapeutic agents between the high‐risk and low‐risk groups. In particular, the MICALL2 gene showed a significant correlation with multiple drugs, suggesting that it may play an important role in regulating glioma drug sensitivity. In addition, we hypothesized that the increase in NETs in the high‐risk group may have led to a specific pattern of immune cell infiltration and may modulate glioma drug sensitivity by affecting drug metabolism, transport or target expression. Of course, we also recognize that other factors such as genetic variants, the composition of the tumor microenvironment, and the patient's systemic immune status may also impact the immune response and drug response in gliomas. Future studies should take these factors into account for a more comprehensive understanding of the immune mechanisms and drug sensitivity in gliomas. In colon adenocarcinoma, MICALL2 play a crucial role in prognosis and immune infiltration levels [51]. Hsu and colleagues' research has found that MICALL2 has a high expression in glioblastoma, compared with LGG, and relevant to survival [52]. Next, we analyzed the expression levels of six prognosis‐related genes between Glioma and Normal samples and found significant differences in ALDH1A3, CHCHD10, and NFIL3. ALDH1A3 is one of the aldehyde dehydrogenase (ALDH) subfamilies that play a key role in different tumor types [53]. ALDH1A3 has been reported in HGG, and inhibiting ALDH1A3‐mediated pathways is a potential treatment strategy for HGG [54]. Li and colleagues also confirmed that ALDH1A3 acts on the differentiation of glioblastoma, and ALDH1A3 is a crucial factor in the prognosis and treatment of glioblastoma patients [55]. According to reports, the level of CHCHD10 is related to mitochondrial‐related disease and neurologic‐type disease [56, 57]. However, without research indicating the impact and mechanism of action in glioma, we found for the first time in our research that CHCHD10 was a prognosis‐related gene with glioma. Nuclear factor interleukin 3 regulated (NFIL3) is an abnormal expression gene in various cancers [58]. However, the mechanism of NFIL3 in glioma is not clear. Interestingly, it's the same as the results of our research that Wu and colleagues have reported that the differentially expressed NFIL3 in glioma subtypes has a better survival prognosis [59]. The expression levels of ALDH1A3, CHCHD10, and NFIL3 in clinical tissue samples and glioma cells found that only NFIL3 had significantly high expression and had the same trend with GSE16011 sets. While ALDH1A3 has both high expression and low expression. The variability of ALDH1A3 expression has been shown in previous studies, where out of 115 glioma tissue specimens, 50 (43.48%) showed low expression of ALDH1A3 and 65 (56.52%) high expression [60]. Moreover, further verified the function of NFIL3 and discovered that knockdown NFIL3 can inhibit glioma cell proliferation, migration and invasion, and promote apoptosis. Previous studies have shown that NFIL3 plays an important role in apoptosis [61, 62].

In conclusion, our results further clarified the function mechanism of NETs‐RGs in glioma and found that six prognosis‐related genes (ACSF2, ALDH1A3, CHCHD10, MGARP, MICALL2, and NFIL3) have better prognosis values for glioma patients. Unfortunately, our analysis revealed that only NFIL3 showed significant differences in expression, further verified the function of NFIL3 in vivo and in vitro, and discovered that knockdown NFIL3 can inhibit glioma cell proliferation, migration and invasion, and promote apoptosis. However, validation of ALDH1A3 and CHCHD10 expression levels yielded different differences. The reason for this discrepancy may be related to the fact that glioma is a complex and variable tumor type with obvious heterogeneous characteristics. This means that not only different glioma samples, but also different regions within the same tumor, may show some differences in gene expression levels. Such differences may also stem from the fact that the samples were taken from different parts of the tumor, the stage, or the type of pathology. Gliomas contain a variety of cell types, such as tumor cells, mesenchymal stromal cells, and immune cells, and these different types of cells may have significant differences in gene expression levels. In addition, the degree of differentiation of tumor cells is also an important factor that influences gene expression. Although we have confirmed the prognostic value of these genes in gliomas using bioinformatics and machine learning algorithms, further validation through more in‐depth and systematic experiments is still needed. We expect to be able to more comprehensively understand the mechanism of these genes in glioma development and provide new ideas and targets for precision therapy and individualized treatment strategies for gliomas.

## Author Contributions

**Xiaobing Guo:** writing – original draft preparation, data curation, formal analysis, validation. **Xiaowen Li:** data curation, formal analysis, validation. **Hengxi Li:** data curation, formal analysis, validation. **Yan Cao:** formal analysis, validation, visualization. **Pengfei Zhang:** visualization, resources. **Ping Li:** conceptualization, writing – review and editing.

## Ethics Statement

The study was approved by the Ethics Committee of Kunming Medical University. The animal experiment received approval from the Experimental Animal Ethics Committee of Yunnan Labreal Biotech Co. Ltd. (IACUC Issue No. PZ20240911).

## Consent

All patients signed an informed consent form.

## Conflicts of Interest

The authors declare no conflicts of interest.

## Supporting information

Supplementary Table 1.

Supplementary Table 2.

Supplementary Table 3.

Supplementary Table 4.

Supplementary Table 5.

Supplementary Table 6.

Supplementary Table 7.

Supplementary Table 8.

Supplementary Table 9.

Supplementary Table 10.

Supplementary Table 11.

Supplementary Table 12.

Supplementary Table 13.

Supplementary Table 14.

Supplementary Table 15.

Supplementary Table 16.

Supplementary Table 17.

Supplementary Table 18.

Supplementary Table 19.

Supplementary Table 20.

Supplementary Table 21.

Supplementary Table 22.

Supplementary Table 23.

Supplementary Table 24.

Supplementary Table 25.

Supplementary Table 26.

Supplementary Table 27.

Supplementary Table 28.

Supplementary Table 29.

Supplementary Table 30.

Supplementary Table 31.

Supplementary Table 32.

Supplementary Table 33.

Supplementary Materials.

## Data Availability

Data are provided within the manuscript or Supporting Information files.
