# Supplementary material for: Identification of Prognostic Values of Neutrophil Extracellular Traps‐Related Genes in Glioma Based on Bioinformatics
Source: Immun Inflamm Dis. 2026 Apr 6;14(4):e70422. doi: 10.1002/iid3.70422 (PMC13053668; doi:10.1002/iid3.70422)
Supplement: Supplementary file 34 — Supplementary Materials. [file IID3-14-e70422-s014.docx]

Supplementary Table 1-2: Information on 276 glioma patients and 8 normal samples in TCGA.

Supplementary Table 3-4: Gene expression substrates and clinical data for glioma patients in TCGA

Supplementary Table 5-6: Clinical information on glioblastoma downloaded from the cbioportal website.

Supplementary Table 7-8: Gene expression matrix and clinical data for 325 glioma patients in the CGGA database.

Supplementary Table 9: Information on the 137 NETs-related genes.

Supplementary Table 10: The differential expression genes between Glioma and Normal samples in GSE16011.

Supplementary Table 11-12: The 57 differentially expressed genes obtained.

Supplementary Table 13: Information on 9 up-regulated genes and 48 down-regulated genes.

Supplementary Table 14: Calculated NETs score.

Supplementary Table 15: Green modules contained 931 genes, and turquoise modules contained 2046 genes

Supplementary Table 16: Information on 6 intersection genes.

Supplementary Table 17: Information on 74 GO biological functions.

Supplementary Table 18-19: Information on 6 survival-related genes.

Supplementary Table 20: Results of the proportional hazards (PH) test.

Supplementary Table 21: The risk model of 6 genes.

Supplementary Table 22: The risk score results.

Supplementary Table 23: The CGGA validation sets to verify the risk model.

Supplementary Table 24-27: Univariate and multivariate Cox independent prognostic analysis.

Supplementary Table 28-29: The result of GSEA analysis.

Supplementary Table 30: The ratio of high- and low-risk group samples in 22 immune infiltration cells.

Supplementary Table 31: The expression levels of 12 immune checkpoint molecules.

Supplementary Table 32: The IC_50_ of 138 common chemotherapy and molecular targeted drugs.

Supplementary Table 33: The drugs with an absolute correlation value >0.9 with prognosis-related genes.
